# Inserción “velamentosa”, encefalopatía hipóxico-isquémica y rehabilitación neurológica: reporte de caso

**DOI:** 10.7705/biomedica.5436

**Published:** 2020-10-09

**Authors:** María José Úsuga, Gloria Alejandra Jaramillo, Valentina Palacio, Sergio Andrés Correa, Juan Camilo Suárez-Escudero

**Affiliations:** 1 Escuela de Ciencias de la Salud, Universidad Pontificia Bolivariana, Medellín, Colombia; 2 Línea de Investigación en Discapacidad, Grupo de Investigación en Salud Pública, Facultad de Medicina, Universidad Pontificia Bolivariana, Medellín, Colombia

**Keywords:** cordón umbilical, hipoxia-isquemia encefálica, hipotermia inducida, rehabilitación neurológica, Umbilical cord, hypoxia-ischemia, brain, hypothermia, induced, neurological rehabilitation

## Abstract

La encefalopatía hipóxico-isquémica es una causa frecuente e importante de daño neurológico en recién nacidos a término y prematuros. Un evento centinela de esta condición es la *vasa previa*, específicamente cuando existe anormalidad de la placenta como la inserción “velamentosa” del cordón umbilical. Algunos reportes evidencian la asociación entre estas dos condiciones, pero son escasos los que dan cuenta del proceso de recuperación y del pronóstico neurológico de los niños afectados por ellas.

Se presenta el caso de un paciente, con antecedentes de inserción “velamentosa” del cordón umbilical y encefalopatía hipóxico-isquémica, que recibió hipotermia terapéutica (*cool cap*). Se describe su proceso de rehabilitación neurológica y se calculó el porcentaje de probabilidad de presentar esta condición frente a la población sin estos factores. El niño tenía cinco años y el puntaje en su prueba de Apgar fue de 0 al minuto y de 2 a los 15 minutos. Desarrolló encefalopatía hipóxico-isquémica grave secundaria a una inserción “velamentosa” del cordón umbilical sin diagnóstico prenatal, con gran compromiso neurológico y multisistémico inicial. El proceso de recuperación incluyó el manejo inicial multidisciplinario en la unidad de cuidados intensivos neonatales y el inicio temprano de habilitación neurológica.

Hoy el niño está escolarizado y en terapia integral, no presenta deficiencias motoras ni sensoriales en el examen físico, aunque la prueba neuropsicológica sugiere un riesgo de trastorno por déficit de atención e hiperactividad. Habitualmente, los niños con encefalopatía hipóxico-isquémica grave presentan discapacidad por deficiencias motoras, cognitivas o conductuales. El haber recibido hipotermia terapéutica y un manejo estructurado de rehabilitación redujo en gran medida las deficiencias esperadas y ha promovido un satisfactorio desarrollo físico y neurológico.

## Abstract (en)

Hypoxic-ischemic encephalopathy is a frequent and important cause of neurological problems in term and preterm newborns. A sentinel event of this entity is the vasa previa, specifically when there is an abnormality of the placenta such as a velamentous cord insertion. Some reports have shown the association between these two entities, but those regarding the recovery process and the neurological prognosis of children with both conditions are scarce.

We present the case of a patient with a history of velamentous cord insertion and hypoxic-ischemic encephalopathy who received therapeutic hypothermia (cool cap). We describe his neurological rehabilitation process and we calculated the percentage of probability of presenting this condition compared to the population without these factors. The patient was a five-year-old boy with an Apgar index at birth equal to zero at one minute and equal to two at fifteen minutes who developed severe hypoxic-ischemic encephalopathy secondary to a velamentous cord insertion without prenatal diagnosis and a marked initial neurological and multisystemic compromise. The recovery process included early multidisciplinary management in the neonatal intensive care unit and a focus on early neurological habilitation.

The patient is currently in school and he undergoes comprehensive therapies; on physical examination, he presents no motor or sensory deficiencies. His neuropsychological test suggests the risk of attention deficit hyperactivity disorder. Children with severe hypoxic-ischemic encephalopathy usually have disabilities due to motor, cognitive, and/or behavioral deficiencies. Having received therapeutic hypothermia and a structured rehabilitation process greatly reduced the expected deficiencies according to prognosis and have promoted satisfactory physical and neurological development.

La encefalopatía hipóxico-isquémica es una causa importante de mortalidad y discapacidad a largo plazo en recién nacidos prematuros y a término (1). Ocurre inmediatamente después del parto por un episodio de asfixia perinatal (2) caracterizado por la presencia, por lo menos, de tres de los siguientes elementos: inicio tardío de la respiración, desaceleraciones tardías en la monitorización fetal o tinción de meconio, puntuación de Apgar por debajo de 7 a los 5 minutos, pH sanguíneo del cordón arterial menor de 7, y daño multiorgánico (3), lo que resulta en deterioro de la capacidad de alerta y el despertar, disminución de la respuesta motora y del tono muscular, alteración de los reflejos y convulsiones (2).

La encefalopatía hipóxico-isquémica ocurre de 3 a 5 de 1.000 nacidos vivos y, aproximadamente, en el 25% de ellos se presenta encefalopatía moderada a grave. La mortalidad reportada es del 10 al 40% de los neonatos afectados y, en promedio, uno de cada tres evidencia cierto grado de retraso en el desarrollo neurológico a largo plazo (4).

Según el grado de compromiso, la encefalopatía hipóxico-isquémica se clasifica en leve, moderada o grave. En las formas leve y moderada, la sintomatología empieza a mejorar en las primeras 72 horas de vida, en tanto que, en la grave, el recién nacido se mantiene en estado de estupor o en coma y con hipotonía marcada. En estos casos, la mortalidad fluctúa entre el 50 y el 75% (2).

Hay eventos (centinelas) en el momento del parto que pueden resultar en asfixia perinatal, entre ellos se destacan la embolia de líquido amniótico, el prolapso del cordón umbilical, la hemorragia fetomaterna, la ruptura uterina, la bradicardia fetal grave y sostenida, el paro cardiorrespiratorio y la *vasa previa* (2). Esta última se produce cuando las membranas que conectan el cordón umbilical y la placenta recubren el orificio cervical interno y se anteponen a la presentación fetal, provocando una hemorragia fetal rápida. Se clasifica en dos tipos: en el tipo 1, se provoca una inserción “velamentosa” del cordón umbilical, es decir, una inserción no central del cordón umbilical, el cual aparece sin la protección de la gelatina de Wharton (5,6). Esto resulta en la exposición de los vasos umbilicales en el orificio cervical debido a su trayecto a través de las membranas antes de alcanzar el disco placentario, con la consecuente hipoperfusión fetal y la acidemia causadas por la laceración a que se ven sometidos.

La inserción “velamentosa” del cordón umbilical tiene una incidencia del 1 al 6% en los embarazos gemelares (6) y una frecuencia del 0,1 al 1,8% en las gestaciones en general. Se asocia con un riesgo de 1,5 a 4 veces mayor de prematuridad, bajo peso y estatura al nacer (5), incluidas complicaciones durante la gestación como bajos puntajes de la prueba de Apgar, restricción del crecimiento fetal y desprendimiento de la placenta (7). La obesidad, el tabaquismo durante la gestación, la placenta previa y baja, y las técnicas de reproducción asistida favorecen su desarrollo (5). Es posible detectarla mediante una ecografía prenatal en la que se observan los vasos umbilicales cruzando la pared uterina antes de ingresar al disco placentario (6).

El objetivo de este informe fue reportar el caso de un paciente con diagnóstico de encefalopatía hipóxico-isquémica grave al nacer, secundaria a una inserción “velamentosa” del cordón umbilical sin diagnóstico prenatal y con gran compromiso neurológico y multisistémico, y se centró en la descripción del proceso de habilitación neurológica multidisciplinario, el cual incluyó el manejo en la unidad de cuidados intensivos neonatales con hipotermia terapéutica (*cool cap*). Además, se determinaron los factores de riesgo y los de protección de la madre del paciente frente a la inserción “velamentosa” del cordón umbilical y, con base en las razones de momios (*odds ratio,* OR) reportadas en la literatura médica, se calculó el porcentaje de probabilidad de presentar esta condición en comparación con la población sin estos factores.

## Caso clínico

Se trató de un paciente de cinco años, fruto del segundo embarazo de 38 semanas más 5 días de una madre de 33 años, con tres controles prenatales, seguimiento ecográfico y gestación normales. Nació por parto espontáneo en vértice con depresión neonatal y presentó un paro cardiorrespiratorio que requirió maniobras de reanimación avanzadas. Su puntaje de Apgar fue de 0 al minuto, 0 a los 5 minutos, 1 a los 10 minutos y 2 a los 15 minutos, con un pesó al nacer de 2.750 g y una talla de 49 cm.

En el examen físico inicial, se registró un soplo mesosistólico de grado II/IV, signos de hipoperfusión, extrema palidez de piel y faneras, ausencia de movimientos espontáneos, y alteración de los reflejos de succión y de Moro. Requirió transfusión de hemoderivados, respiración mecánica invasiva por ocho días, oxigenoterapia por cánula nasal por 13 días, y se le sometió al protocolo de hipotermia terapéutica con la temperatura central a 34 ºC y la del gorro a 14 ºC durante los primeros tres días de vida.

Según la clasificación de Sarnat, la encefalopatía hipóxica del paciente fue grave (Sarnat III), pues en los primeros tres días posteriores al nacimiento, el niño presentó convulsiones, hipotonía global y ausencia de reflejos primitivos (2). En los exámenes iniciales de laboratorio, se reportó acidosis metabólica en sangre del cordón umbilical, con pH de 6,94, PCO_2_ de 28,4 mm Hg, PO_2_ de 214,3 mm Hg saturación del 98,8%, HCO_2_ de 6,6 mm Hg y exceso de base de -26,4 mEq/L. En el ionograma, se reportaron concentraciones de fósforo de 5 mg/dl, de potasio de 5 mmol/L, de magnesio de 1,8 mg/dl, de calcio de 8,8 mg/dl, de amonio de 41 mg/dl, y de ácido láctico de 6,3 mmol/L. Se detectó alteración en las pruebas de coagulación (TPT=76 s y TP=17 s), así como transaminasas elevadas (ALT=86 U/L; AST=207 U/L) e hiperglucemia (336 mg/dl).

En el examen macroscópico de la placenta se encontró inserción “velamentosa” del cordón umbilical sin diagnóstico prenatal, la cual causó abundante sangrado y anemia aguda (hemoglobina: 8,9 g/dl; hematocrito: 27,7%), y desembocó en una encefalopatía hipóxico-isquémica grave del neonato secundaria a asfixia perinatal grave como diagnóstico principal.

Al segundo día del nacimiento, el electroencefalograma (EEG) era anormal pues mostró cuatro episodios convulsivos, dos de ellos con correlación clínica, los otros dos prolongados y con actividad semicontinua, por lo que se diagnosticaron crisis eléctricas semicontinuas. Además, en las primeras horas de vida, el niño presentó hepatitis y miocarditis hipóxicas, así como hemorragia pulmonar y, en el ecocardiograma, signos de hipertensión pulmonar leve y de ventrículo izquierdo hiperdinámico.

**Figura 1:**
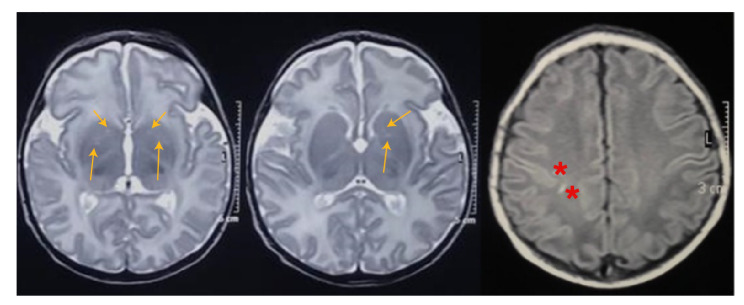
En la secuencia potenciada en T2 (primeras dos imágenes), se sugiere la presencia de secuelas por encefalopatía hipóxico-isquémica en los núcleos lenticulares (flechas). En la secuencia T1 (tercera imagen), se evidencian señales puntiformes hiperintensas subcorticales en los lóbulos parietal y frontal derechos (asterisco).

Otros hallazgos en el curso de la hospitalización incluyeron la disminución de la amplitud y de la frecuencia de movimientos espontáneos, falta de fijación y seguimiento visual, ausencia del reflejo nauseoso, succión lenta y descoordinada e hipotonía generalizada persistente. En la resonancia magnética realizada a los 12 días del nacimiento, se evidenciaron lesiones de los ganglios basales y subcorticales derechas secundarias a hipoxia sin lesión hemorrágica (figura 1), en tanto que, en el estudio polisomnográfico basal, se registró actividad epileptiforme focal frontal izquierda y temporal bilateral. También, se reportaron potenciales evocados visuales anormales por ausencia de la banda P, sugestivos de lesión prequiasmática o cortical.

En cuanto a los reportes del desarrollo psicomotor del paciente, se encontró sedestación a los siete meses, gateo a los 8 meses y marcha a los 12 meses. El desarrollo del lenguaje ha sido adecuado, y las habilidades comunicativas y las relaciones interpersonales han mejorado con la edad. El proceso de habilitación incluyó el manejo multidisciplinario enfocado en la habilitación neurológica y el niño recibió rehabilitación física con el método de Vöjta a partir de los 36 días de vida. Inicialmente, se observó una alteración grave en la reactividad postural, la dinámica de los reflejos primitivos, el tono muscular y la hiperreflexia osteotendinosa. Al final de la terapia, presentaba tono muscular normal, movilización de las cuatro extremidades, control del tronco en sedestación, marcha estable con apoyo bipodal, y fuerza muscular y movimientos normales de las extremidades.

Se le hicieron sesiones de fonoaudiología desde el nacimiento por ausencia del reflejo de succión, disfagia preoral y oral, y riesgo de broncoaspiración. Se realizaron 25 sesiones centradas en la estimulación de los músculos implicados en la succión y la deglución con lo que, finalmente, logró una succión efectiva con adecuada fuerza, continuidad y deglución. Desde los dos años, asistió semanalmente a terapia de lenguaje durante 19 sesiones por trastorno específico de la pronunciación y pudo seguir el proceso fonético acorde con su edad.

El niño también recibió terapia ocupacional con énfasis en la integración sensorial durante un año y nueve meses, con lo que hubo una mejoría del 80% en las respuestas vestíbulo-motoras y el reconocimiento de su esquema corporal.

La prueba neuropsicológica que se le hizo a los cinco años en un centro de referencia de Medellín reflejó una capacidad intelectual general de 84 puntos (rango normal bajo). En el aspecto emocional se han detectado rasgos de conductas atípicas con comportamientos no acordes con determinados contextos y en el hogar. En el contexto académico, lo perciben con depresión y conducta agresiva, aunque sus habilidades adaptativas se ubican en el rango promedio para la edad; las destrezas sociales y comunicativas, las de la vida personal y las de la vida en comunidad corresponden al rango promedio bajo, pero sin cumplir con los criterios de discapacidad.

El niño presenta dificultades en cuanto a la memoria de trabajo y la espacial, la verbal auditiva inmediata, la denominación e integración visual, y la secuenciación. Posee habilidades para la velocidad de procesamiento, la memoria verbal auditiva a corto plazo, el lenguaje comprensivo, los dispositivos básicos de atención sostenida, alternante y selectiva, la asociación de conceptos, la identificación de letras y palabras, y los dispositivos básicos de cálculo, así como para el aprendizaje por asociación. Con base en estos elementos, la impresión diagnóstica es sugestiva –en riesgo– de un trastorno por déficit de atención e hiperactividad de tipo no especificado.

Actualmente, el niño tiene seis años, está escolarizado (primero de primaria), asiste a terapias de fonoaudiología, conductivo-conductuales, ocupacionales y de psicología. Tras el resultado de la prueba neuropsicológica le fue suspendido el montelukast, única medicación que recibía hasta ese momento. En el examen físico no se reportó dimorfismo ni en el rostro ni en las extremidades, los nervios craneales no presentaban alteraciones, el corazón latía con ritmo, no se evidenciaron soplos y los pulmones estaban bien ventilados, los testículos bien descendidos, sus reflejos eran normales, estaba eutónico y no había reflejos patológicos. Presentaba una fuerza de 5/5 en las cuatro extremidades, y la medición, la diadococinesia y el movimiento de las manos eran normales. El niño nombra, repite y comprende órdenes; su fonación es normal y su marcha y equilibrio no presentan alteraciones.

## Discusión

Las anormalidades del cordón umbilical pueden ocasionar complicaciones durante el embarazo y el trabajo de parto, y causar encefalopatía hipóxico-isquémica en el neonato debido a la ruptura de los vasos y la pérdida sanguínea, lo cual sucedió en el paciente de este caso. Según los informes publicados, aproximadamente, el 39% de los nacidos vivos con diagnóstico de encefalopatía hipóxico-isquémica presentan inserción anormal del cordón umbilical, ya sea marginal o “velamentosa”, en comparación con el 7% de los nacidos vivos sanos (4).

El pronóstico de la encefalopatía hipóxico-isquémica en neonatos se determina en gran medida por la gravedad y la extensión del daño cerebral. Los niños con una forma leve tienen poca probabilidad de experimentar consecuencias graves y la mayoría tienen un desarrollo normal a largo plazo; aquellos con una forma moderada tienen entre el 30 y el 50% de riesgo de parálisis cerebral como complicación, y los niños con afectación grave, como el presente, presentan una elevada tasa de mortalidad o un gran riesgo de quedar con discapacidades graves, siendo una de las principales la discapacidad por deficiencias motoras, cognitivas o conductuales. Entre las deficiencias motoras causadas por la forma grave de este tipo de encefalopatía, sobresale la tetraplejia (8-10). En el presente caso no se produjo ningún déficit motor permanente.

Además de los hallazgos clínicos, las anormalidades en las imágenes cerebrales y en el electroencefalograma también permiten predecir las deficiencias neurológicas a largo plazo en estos pacientes; dichas alteraciones se asocian con resultados adversos y una evolución clínica deficiente, independientemente de la gravedad de la encefalopatía hipóxico-isquémica o el uso de la hipotermia terapéutica (11-14).

Acorde a la presencia de hallazgos estructurales, el paciente presentó anormalidades en las neuroimágenes (lesiones en los ganglios basales y subcorticales derechas secundarias a la hipoxia). Asimismo, se reportó un electroencefalograma anormal en concordancia con la aparición de convulsiones, lo que se ha asociado con resultados adversos en múltiples estudios (15,16). Sin embargo, en este paciente no hubo convulsiones después del periodo neonatal.

En los últimos años ha cobrado una gran importancia el uso de la hipotermia terapéutica, la cual se considera el tratamiento de referencia de la encefalopatía hipóxico-isquémica (17). En los pacientes nacidos con 36 semanas o más de gestación y encefalopatía hipóxico-isquémica moderada a grave, dicho tratamiento debe iniciarse en las primeras seis horas tras el nacimiento y continuarse durante 72 horas. Además, deben cumplir alguna de las siguientes condiciones: acidosis grave dentro de la primera hora de nacimiento, puntaje de Apgar de 5 o menos a los 10 minutos o reanimación durante 10 minutos o más tras el nacimiento (18).

En el presente paciente se cumplían los criterios de elegibilidad para este tipo de terapia, cuyo uso tiene un efecto positivo en cuanto a la aparición de deficiencias capaces de generar discapacidad entre los 18 y los 24 meses de edad (19) y aumenta la supervivencia (20). Además, los recién nacidos que presentan circunstancias perinatales centinelas como la ruptura del cordón umbilical, entre otras, y reciben la hipotermia suelen tener menos lesiones (21). El uso de la hipotermia terapéutica en este paciente fue fundamental para mejorar el pronóstico físico, cognitivo y sensorial, y potenciar las acciones de habilitación multidisciplinaria implementadas de manera temprana.

Los pacientes que sobreviven a las lesiones cerebrales tienen un amplio espectro de deficiencias que incluye desde compromisos leves, como los problemas de atención, memoria, comportamiento y trastornos del aprendizaje (22), hasta otros más graves e incapacitantes como epilepsia, discapacidad visual, trastornos cognitivos o del desarrollo y parálisis cerebral. También, se han descrito diversos grados de discapacidad motora (23). En un estudio de 110 sobrevivientes de encefalopatía hipóxico-isquémica, se observó que más del 25% de los niños de seis a siete años de edad tenía puntuaciones de coeficiente intelectual por debajo del valor normal (9).

En el presente caso, el desarrollo psicomotor en cuanto al gateo, la sedestación y la bipedestación fue adecuado para la edad, y hoy su desarrollo del lenguaje, sus patrones de comportamiento y su desarrollo en el ambiente escolar son satisfactorios. Todo ello se logró mediante el acompañamiento multidisciplinario de profesionales en estas áreas y un programa estructurado de habilitación neurológica enfocado en terapias del lenguaje, física, de la deglución y ocupacional con énfasis en la integración sensorial. Todo esto ha contribuido a reducir las deficiencias esperadas según el pronóstico y ha permitido que el paciente tenga un satisfactorio desarrollo físico y neurológico.

Los factores de riesgo conocidos para la encefalopatía hipóxico-isquémica incluyen una edad materna de 35 o más años, preeclampsia, corioamnionitis, restricción del crecimiento uterino y presentación de alguno de los eventos centinela (24), lo que es una causa rara de esta enfermedad, pero que en el caso expuesto fue el único factor de riesgo presente.

Tras realizar una revisión de la literatura sobre los factores de riesgo para la presentación de inserción “velamentosa” del cordón umbilical, se compararon las razones de momios (*odds ratio*, OR) con los presentados por la madre del paciente para, así, proponer un porcentaje de riesgo para la presentación de esta condición ((25,26)) (cuadro 1).

**Cuadro 1:** Factores de riesgo y de protección considerados en este caso

**Criterio**	*Odds Ratio* en la literatura*	*Odds Ratio* calculado**
**Factores de riesgo**		
Sangrado vaginal	1,98 (1,84-2,23)	66,4 (64,7-69)
Asma materno	1,23 (1,12,1,34)	55,1 (52,^8^-57,^2^)
Edad igual o mayor de 34 años	1,22 (1,06-1,4)	54,9 (51,^4^-58,^3^)
**Factores protectors**		
Partos multiples	0,78 (0,66-0,92)	18 (7,4-25,3)

Factores de riesgo

* aumenta x veces la posibilidad de presentar una inserción velamentosa (sic.) del cordón umbilical; ** aumenta en x% la probabilidad de presentar inserción velamentosa (sic.) del cordón umbilical frente a las mujeres que no la presentan.

Factores de protección

* disminuye la posibilidad de presentar inserción velamentosa del cordón umbilical en x veces

** disminuye x% la probabilidad de presentar inserción velamentosa del cordón umbilical frente a quienes no la presenten

En diversos estudios se sugiere el seguimiento constante de los niños con encefalopatía hipóxico-isquémica hasta la edad escolar, ya que se pueden presentar alteraciones después de los cinco años: dificultades cognitivas o conductuales asociadas con las lesiones cerebrales tras una encefalopatía neonatal, las cuales se reflejan más en el cuerpo estriado y en el hipocampo (23). En el caso expuesto, el paciente está siendo estudiado para establecer el diagnóstico de trastorno por déficit de atención e hiperactividad, y posee lesiones en los núcleos lenticulares.

En Colombia, se reportó una mortalidad por asfixia perinatal del 7% en el 2008 (27) y, en el 2017, en un estudio de 8.837 niños atendidos entre el 2007 y el 2011 en un hospital universitario de Bogotá, se reportó que 24 presentaron asfixia perinatal, con una tasa de mortalidad global del 21% (27). En otro estudio en Antioquia en el 2019, se reportaron 3.901 muertes perinatales en fetos con más de 22 semanas de gestación o 500 g y hasta los 28 días de vida por hipoxia antes y durante el parto (28). En otro estudio en el que se evaluaron las características clínicas de los pacientes con encefalopatía hipóxico-isquémica sometidos a hipotermia cerebral, se observó que el 90% tenía algún grado de encefalopatía en el momento del egreso, y aquellos pacientes con un puntaje de Apgar bajo a los 10 minutos y acidemia presentaron mayor mortalidad (29). Al parecer, la mortalidad está relacionada con la edad de inicio del protocolo de hipotermia, la alteración de las enzimas hepáticas y cardiacas, y los valores de las plaquetas y del lactato (30). En un estudio en Cali, se reportaron como factores de riesgo para desarrollar asfixia perinatal, los siguientes: desprendimiento prematuro de la placenta, trabajo de parto con expulsión prolongada, no haber usado oxitocina, pocos controles prenatales y los factores sociales (por ejemplo, el ser madre soltera) (31).

Tras una búsqueda en la literatura médica, no se encontraron reportes de un caso igual al expuesto, en el que se obtuvo una recuperación neurológica satisfactoria a largo plazo mediante un programa de rehabilitación multidisciplinario en un paciente con encefalopatía hipóxico-isquémica grave secundaria a inserción “velamentosa” del cordón umbilical, lo cual contradice los pronósticos usuales. Es importante tener en cuenta que en varios estudios se ha reportado la asociación entre estas dos condiciones, comúnmente, en niños con diagnóstico de parálisis cerebral o muerte (4).

## References

[1] Lemyre B, Chau V. Hypothermia for newborns with hypoxic-ischemic encephalopathy. Paediatr Child Health. 2018 Jul;23(4):285–91. 10.1093/pch/pxy028 30657134 PMC6007306

[2] García-Alix A, Martínez M, Arnaez J, Valverde E, Quero J. Asfixia intraparto y encefalopatía hipóxico-isquémica. Asfixia intraparto y encefalopatía hipóxico-isquémica: protocolos diagnósticos terapéuticos de la AEP: Neonatología. Madrid: Asociación Española de Pediatría; 2008. p. 242-52.

[3] Graham EM, Ruis KA, Hartman AL, Northington FJ, Fox HE. A systematic review of the role of intrapartum hypoxia-ischemia in the causation of neonatal encephalopathy. Am J Obstet Gynecol. 2008 Dec;199(6):587–95. 10.1016/j.ajog.2008.06.094 19084096

[4] Nasiell J, Papadogiannakis N, Löf E, Elofsson F, Hallberg B. Hypoxic ischemic encephalopathy in newborns linked to placental and umbilical cord abnormalities. J Matern Fetal Neonatal Med. 2016 Mar;29(5):721–6. 10.3109/14767058.2015.1015984 25714479

[5] Wiedaseck S, Monchek R. Placental and cord insertion pathologies: screening, diagnosis, and management. J Midwifery Womens Health. 2014 May-Jun;59(3):328–35. 10.1111/jmwh.12189 24751147

[6] Cunningham FG, Leveno KJ, Bloom SL, Dashe JS, Hoffman BL, Casey BM, et al. Placental abnormalities. Williams Obstetrics. 25^th^. edition. New York, NY: McGraw-Hill Education; 2018. p. 111-23.

[7] de Los Reyes S, Henderson J, Eke AC. A systematic review and meta-analysis of velamentous cord insertion among singleton pregnancies and the risk of preterm delivery. Int J Gynaecol Obstet. 2018 Jul;142(1):9–14. 10.1002/ijgo.12489 29572823 PMC9233492

[8] Thornberg E, Thiringer K, Odeback A, Milsom I. Birth asphyxia: incidence, clinical course and outcome in a Swedish population. Acta Paediatr. 1995 Aug;84(8):927–32. 10.1111/j.1651-2227.1995.tb13794.x 7488819

[9] Pappas A, Shankaran S, McDonald SA, Vohr BR, Hintz SR, Ehrenkranz RA, et al.; Hypothermia Extended Follow-up Subcommittee of the Eunice Kennedy Shriver NICHD Neonatal Research Network. Cognitive outcomes after neonatal encephalopathy. Pediatrics. 2015 Mar;135(3):e624–34. 10.1542/peds.2014-1566 25713280 PMC4338321

[10] Robertson C, Finer N. Term infants with hypoxic-ischemic encephalopathy: outcome at 3.5 years. Dev Med Child Neurol. 1985 Aug;27(4):473–84. 10.1111/j.1469-8749.1985.tb04571.x 4029517

[11] Walsh BH, Neil J, Morey J, Yang E, Silvera MV, Inder TE, et al. The frequency and severity of magnetic resonance imaging abnormalities in infants with mild neonatal encephalopathy. J Pediatr. 2017 Aug;187:26–33.e1. 10.1016/j.jpeds.2017.03.065 28479101 PMC5533615

[12] Holmes GL, Lombroso CT. Prognostic value of background patterns in the neonatal EEG. J Clin Neurophysiol. 1993 Jul;10(3):323–52. 10.1097/00004691-199307000-00008 8408599

[13] Awal MA, Lai MM, Azemi G, Boashash B, Colditz PB. EEG background features that predict outcome in term neonates with hypoxic ischaemic encephalopathy: A structured review. Clin Neurophysiol. 2016 Jan;127(1):285–96. 10.1016/j.clinph.2015.05.018 26105684

[14] Jain SV, Mathur A, Srinivasakumar P, Wallendorf M, Culver JP, Zempel JM. Prediction of neonatal seizures in hypoxic-ischemic encephalopathy using electroencephalograph power analyses. Pediatr Neurol. 2017 Feb;67:64–70.e2. 10.1016/j.pediatrneurol.2016.10.019 28062149

[15] Kharoshankaya L, Stevenson NJ, Livingstone V, Murray DM, Murphy BP, Ahearne CE, et al. Seizure burden and neurodevelopmental outcome in neonates with hypoxic-ischemic encephalopathy. Dev Med Child Neurol. 2016 Dec;58(12):1242–8. 10.1111/dmcn.13215 27595841 PMC5214689

[16] Fitzgerald MP, Massey SL, Fung FW, Kessler SK, Abend NS. High electroencephalographic seizure exposure is associated with unfavorable outcomes in neonates with hypoxic-ischemic encephalopathy. Seizure. 2018 Oct;61:221–6. 10.1016/j.seizure.2018.09.003 30227341 PMC6168337

[17] Pisani F, Spagnoli C. Monitoring of newborns at high risk for brain injury. Ital J Pediatr. 2016 May;42(1):48. 10.1186/s13052-016-0261-8 27180227 PMC4867092

[18] Chiang MC, Jong YJ, Lin CH. Therapeutic hypothermia for neonates with hypoxic ischemic encephalopathy. Pediatr Neonatol. 2017 Dec;58(6):475–83. 10.1016/j.pedneo.2016.11.001 28416250

[19] Nariño O, Vera C, Carvajal J. Hipotermia corporal total en neonatos con encefalopatía hipóxico-isquémica (1). Rev Chil Obstet Ginecol. 2006;71:73–5. 10.4067/S0717-75262006000100013

[20] Azzopardi DV, Strohm B, Edwards AD, Dyet L, Halliday HL, Juszczak E, et al.; TOBY Study Group. Moderate hypothermia to treat perinatal asphyxial encephalopathy. N Engl J Med. 2009 Oct;361(14):1349–58. 10.1056/NEJMoa0900854 19797281

[21] Shankaran S, Laptook AR, McDonald SA, Hintz SR, Barnes PD, Das A, et al.; Eunice Kennedy Shriver National Institute of Child Health, and Human Development Neonatal Research Network. Acute perinatal sentinel events, neonatal brain injury pattern and outcome of infants undergoing a trial of hypothermia for neonatal hypoxic-ischemic encephalopathy. J Pediatr. 2017 Jan;180:275–278.e2. 10.1016/j.jpeds.2016.09.026 27776752 PMC5183477

[22] Hayes BC, Doherty E, Grehan A, Madigan C, McGarvey C, Mulvany S, et al. Neurodevelopmental outcome in survivors of hypoxic ischemic encephalopathy without cerebral palsy. Eur J Pediatr. 2018 Jan;177(1):19–32. 10.1007/s00431-017-3028-3 29063960

[23] van Schie PE, Schijns J, Becher JG, Barkhof F, van Weissenbruch MM, Vermeulen RJ. Long-term motor and behavioral outcome after perinatal hypoxic-ischemic encephalopathy. Eur J Paediatr Neurol. 2015 May;19(3):354–9. 10.1016/j.ejpn.2015.01.005 25683783

[24] Parker SJ, Kuzniewicz M, Niki H, Wu YW. Antenatal and intrapartum risk factors for hypoxic-ischemic encephalopathy in a US birth cohort. J Pediatr. 2018 Dec;203:163–9. 10.1016/j.jpeds.2018.08.028 30270166

[25] Ebbing C, Kiserud T, Johnsen SL, Albrechtsen S, Rasmussen S. Prevalence, risk factors and outcomes of velamentous and marginal cord insertions: a population-based study of 634,741 pregnancies. PLoS One. 2013 Jul;8(7):e70380. 10.1371/journal.pone.0070380 23936197 PMC3728211

[26] Räisänen S, Georgiadis L, Harju M, Keski-Nisula L, Heinonen S. Risk factors and adverse pregnancy outcomes among births affected by velamentous umbilical cord insertion: a retrospective population-based register study. Eur J Obstet Gynecol Reprod Biol. 2012 Dec;165(2):231–4. 10.1016/j.ejogrb.2012.08.021 22944380

[27] Del Riesgo-Prendes L, Salamanca-Matta AL, Monterrey-Gutiérrez PA, Bermúdez-Hernández PA, Vélez JL, Suárez-Rodríguez G. Hipoxia perinatal en el Hospital Mederi de Bogotá: comportamiento en los años 2007 a 2011. Rev Salud Publica (Bogota). 2017 May-Jun;19(3):332–9. 10.15446/rsap.v19n3.65204 30183937

[28] Zuleta-Tobón JJ, Salazar-Barrientos M. Aplicación del Sistema de Clasificación Internacional de Enfermedades para la Mortalidad Perinatal CIE-MP a partir de registros vitales para clasificar las muertes perinatales en Antioquia, Colombia. Rev Colomb Obstet Ginecol. 2019;70:228–42. 10.18597/rcog.3406 32142238

[29] Vargas-Vaca Y, Devia C, Bertolotto AM, Suárez-Obando F. Caracterización de los recién nacidos con asfixia perinatal moderada o severa manejados con hipotermia cerebral selectiva en la Unidad de Recién Nacidos del Hospital Universitario San Ignacio desde junio de 2015 hasta marzo de 2017. Univ Med. 2019;60:4–13. 10.11144/Javeriana.umed60-4.crna

[30] Manotas H, Troncoso G, Sánchez J, Molina G. Descripción de una cohorte de pacientes neonatos con diagnóstico de asfixia perinatal, tratados con hipotermia terapéutica. 2017. Perinatol Reprod Hum. 2018;32:70–7. 10.1016/j.rprh.2018.07.001

[31] Torres-Muñoz J, Rojas C, Mendoza-Urbano D, Marín-Cuero D, Orobio S, Echandía C. Factores de riesgo asociados con el desarrollo de asfixia perinatal en neonatos en el Hospital Universitario del Valle, Cali, Colombia, 2010-2011. Biomédica. 2017;37:51–6. 10.7705/biomedica.v37i1.2844 28527266

